# GIS Partial Discharge Pattern Recognition Based on a Novel Convolutional Neural Networks and Long Short-Term Memory

**DOI:** 10.3390/e23060774

**Published:** 2021-06-18

**Authors:** Tingliang Liu, Jing Yan, Yanxin Wang, Yifan Xu, Yiming Zhao

**Affiliations:** State Key Laboratory of Electrical Insulation and Power Equipment, Xi’an Jiaotong University, Xi’an 710049, China; liutingliang12@stu.xjtu.edu.cn (T.L.); yxwang1227@gmail.com (Y.W.); xyf9894326@stu.xjtu.edu.cn (Y.X.); zhaoyiming96@stu.xjtu.edu.cn (Y.Z.)

**Keywords:** convolutional neural network, long short-term memory, partial discharge, pattern recognition

## Abstract

Distinguishing the types of partial discharge (PD) caused by different insulation defects in gas-insulated switchgear (GIS) is a great challenge in the power industry, and improving the recognition accuracy of the relevant models is one of the key problems. In this paper, a convolutional neural network and long short-term memory (CNN-LSTM) model is proposed, which can effectively extract and utilize the spatiotemporal characteristics of PD input signals. First, the spatial characteristics of higher-level PD signals can be obtained through the CNN network, but because CNN is a deep feedforward neural network, it does not have the ability to process time-series data. The PD voltage signal is related to the time dimension, so LSTM saves and analyzes the previous voltage signal information, realizes the modeling of the time dependence of the data, and improves the accuracy of the PD signal pattern recognition. Finally, the pattern recognition results based on CNN-LSTM are given and compared with those based on other traditional analysis methods. The results show that the pattern recognition rate of this method is the highest, with an average of 97.9%, and its overall accuracy is better than that of other traditional analysis methods. The CNN-LSTM model provides a reliable reference for GIS PD diagnosis.

## 1. Introduction

Gas-insulated switchgear (GIS) is widely used in power systems. Once insulation defects occur, they directly affect the normal operation of an entire power system. Therefore, the timely discovery and identification of the partial discharge (PD) caused by different types of insulation defects in GIS is of great significance [1]. However, it is also difficult for the most experienced experts to distinguish certain types of PD signals because they have very similar characteristics. In order to improve the accuracy of partial discharge pattern recognition, algorithms such as decision trees (DT), back propagation neural networks (BPNN), support vector machines (SVM), and other deep learning algorithms are adopted for different pattern recognition of PD signals [2]. Among many deep learning methods, convolutional neural networks (CNN) have received widespread attention due to their powerful advantages in automatically extracting the spatial features of images, while long short-term memory (LSTM) has been widely used due to its powerful processing capabilities for time series.

In recent years, the intelligent diagnosis method using CNN for pattern recognition has achieved excellent results in GIS PD pattern recognition and classification due to its powerful automatic feature extraction and classification capabilities. Based on a CNN, a deep learning method for PD pattern recognition is proposed in [3]. The factors affecting the accuracy of pattern recognition, including the network layer, convolution kernel size, and activation function, are discussed. Compared with the traditional machine learning methods, the results show that the accuracy of the CNN method is better than that of the traditional methods. In this paper, a spectrum-optimization CNN based on cross layer feature fusion is proposed. First, the variational modal decomposition (VMD) algorithm is used to decompose the PD signal into multiple components, and the simulation is performed to obtain the time spectrum; second, a cross-layer feature fusion optimization CNN is constructed, and its recognition accuracy is obviously better than that of the existing recognition methods.

Recurrent neural networks (RNN), especially those that use hidden units of LSTM, are a powerful and recent research hotspot model that can be used to learn from sequence data. In [4], a fault in a railway track circuit was diagnosed by generating an LSTM network model, and 99.7% of the test input sequences were correctly classified. By comparison, LSTM has better performance in fault detection and identification of railway track circuit than CNN. In [5,6], a novel detection method based on a deep autoencoder was proposed, which performed the unsupervised diagnosis of motor faults and evaluated three different autoencoder architectures: the multilayer perceptron (MLP) auto-encoder, CNN auto-encoder, and cyclic auto-encoder composed of an LSTM unit. In view of the above considerations, both CNN and LSTM provide satisfactory fault diagnosis pattern recognition results in a short period of time [6]. Therefore, it is foreseeable that a reasonable integration of CNN and LSTM will further reduce the classification errors. Therefore, this paper combines the advantages of CNN and LSTM and proposes a new a convolutional neural network and long short-term memory (CNN-LSTM) network for PD pattern recognition. Compared with traditional PD pattern recognition, to the best of our knowledge, this is a brand-new pattern recognition method. The main contributions of this paper are as follows:

(1) Combining the advantages of CNN and LSTM, a CNN-LSTM hybrid network is proposed for the pattern recognition of PD, which achieved excellent results.

(2) In the proposed CNN-LSTM model, CNN can automatically extract features as inputs. Combined with the advantages of LSTM in the analysis of data dependency and time dynamics, a new method of PD signal feature extraction is constructed, using CNN to extract spatial features and LSTM to extract temporal features.

(3) The feasibility of the model is verified through experiments and compared with other traditional classification methods. The results show that the average recognition accuracy of the CNN-LSTM network proposed in this article reached 98%, which is better than the accuracy of other traditional methods [7,8,9].

The structure of the remainder of this paper is as follows: Section 2 introduces the basics of the CNN and LSTM deep learning algorithms; Section 3 introduces the overall structure of the proposed CNN-LSTM in detail; Section 4 conducts an experimental verification of the proposed CNN-LSTM network and compares it with a variety of traditional algorithms; and Section 5 provides a summary.

## 2. Basic Theory

### 2.1. Convolutional Neural Network

**Figure 1:**
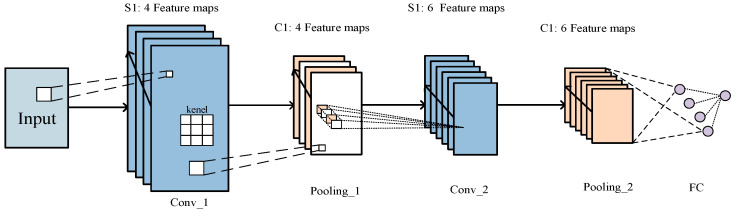
The basic structure of convolutional neural networks (CNN).

CNN is an efficient pattern-recognition method developed in recent years. It is widely used in GIS PD pattern recognition and classification due to its powerful automatic feature capture ability. Generally, a CNN is mainly composed of an input layer, a convolutional layer, a pooling layer, and a fully connected (FC) layer. The local connection and parameter sharing in the CNN reduce the number of parameters, greatly reduce the training complexity, and reduce overfitting. At the same time, its weight-sharing also makes the CNN tolerant of translations, while the down-sampling in the pooling layer further reduces the output parameters and makes the model tolerant to mild deformations, which improves the generalization ability of the model [10,11,12]. As shown in Figure 1, in each feature extraction layer, the feature map performs convolution calculation on multiple convolution kernels, and the feature extraction layers are connected by deviation calculation, activation function, and pooling operation. The operation of each feature extraction layer can be expressed as

(1) $$x_{j}^{l}=f(\sum_{i\in M_{j}}x_{i}^{l-1\;}{*\;k}_{\mathrm{ij}}^{l}{+b}_{j}^{l}),$$

where $x_{i}^{l-1}$ represents the input of the lth layer, $x_{j}^{l}$ represents the output of the lth layer, and b represents the weight, $M_{j}$ represents the jth option in the feature map, and $k_{\mathrm{ij}}^{l}$ is the convolution kernel between the j-th feature map of the l-th layer and the i-th feature map of the (l−1) layer.

For the convolution calculation, the input feature map is outputted through the convolution calculation and activation function, which can be expressed as:

(2) $$M_{j}^{l}{=\sigma (M}_{j}^{l-1}{\;*\;W}_{j}^{l-1}{+b}_{j}^{l-1}),$$

where σ represents the activation function, $W_{j}^{l-1}$ represents the weight matrix of the “j”th layer, and $b_{j}^{l-1}$ is the bias vector of the jth layer.

The result of the pooling layer can be obtained by Formula (3).

(3) $$M_{j}^{l}{=\mathrm{pooling}(M}_{j}^{l-1}),$$

In the Formula (3), pooling means pooling operation. Commonly used pooling methods include maximum pooling, mean pooling, and random pooling.

The fully connected layer obtains the output through the weighted summation operation of the feature map of the previous layer and the activation function response, which can be expressed as

(4) $$M_{j}^{l}{=\sigma (M}_{j}^{l-1}\cdot W_{j}^{l-1}{+b}_{j}^{l-1}).$$

### 2.2. Long and Short-Term Memory

**Figure 2:**
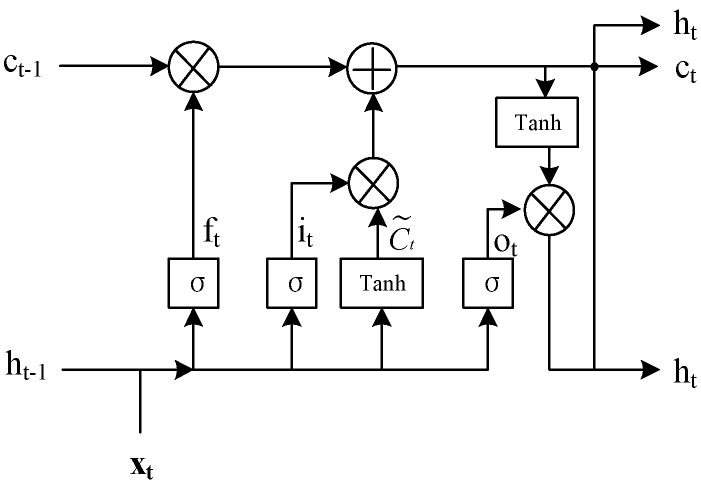
The basic structure of the long short-term memory (LSTM) cell.

As a special form of RNN network, LSTM network has the ability to process time series. It can store previous data in its storage unit and is very effective in predicting time series data. Using the LSTM network can alleviate the gradient disappearance and explosion problems found in RNN. The LSTM network consists of four basic components: unit, input gate, output gate, and forget gate. This information is sent by the unit at random time intervals. The gate tracks the flow of input and output data from the unit. The forgetting gate determines how many cell states from the previous moment are retained in the current moment. The input gate determines the amount of current network’s input stored in the unit state. The output gate controls how much of the unit state is outputted to the current output value of the LSTM [13,14,15]. The basic configuration of the LSTM cell is shown in Figure 2.

According to the calculation sequence of the algorithm, the forward calculation formula of the LSTM is shown in Formula (5). In the formula, $x_{t}$ represents the input, $f_{t}$ represents the forget gate, $o_{t}$ represents the output gate, $i_{t}$ represents the input gate, $h_{t}$ represents the hidden layer state, $C_{t}$ represents the cell state, $\tilde{C_{t}}$ represents the candidate value of the cell state, σ represents the activation of each gate, W represents the weight, and b represents the deviation. For example, in the calculation formula of the forget gate, $W_{f}$ is the weight matrix of the forgetting gate, [$h_{t-1}{,x}_{t}$] is the connection between two vectors in a longer vector, $b_{f}$ is the bias term of the forgetting gate, and σ is the sigmoid function.

The node output of the LSTM cell is calculated as follows:

(5) $$\{\begin{array}{c} i_{t}=\sigma \left(W_{i}\cdot \left[h_{t-1},x_{t}\right]+b_{i}\right)\\ f_{t}=\sigma \left(W_{f}\cdot \left[h_{t-1},x_{t}\right]+b_{f}\right)\\ \tilde{C_{t}}=\tanh\left(W_{c}\cdot \left[h_{t-1},x_{t}\right]+b_{c}\right)\\ C_{t}=f_{t}*C_{t-1}+i_{t}*\tilde{C_{t}}\\ o_{t}=\sigma \left(W_{\sigma }\cdot \left[h_{t-1},x_{t}\right]+b_{o}\right)\\ h_{t}=o_{t}*\tanh\left(C_{t}\right) \end{array}$$

## 3. Proposed Method

CNN can perform feature extraction and data processing adaptively and reduce the dimensionality through convolution and merging, and it has a better generalization ability than traditional features. In PD fault diagnosis, the signal is usually converted into a time-domain diagram or a time-frequency diagram and then processed by a two-dimensional (2D) CNN. However, the 2D convolution operation can only extract spatial features, while ignoring the time features of the signal, resulting in the poor performance of the above model under complex interference. Considering the characteristics of the PD signal, the 2D convolution operation is used to convolve along the time axis of the signal to extract the features in order to ensure feature extraction while retaining the time features [16,17].

**Figure 3:**
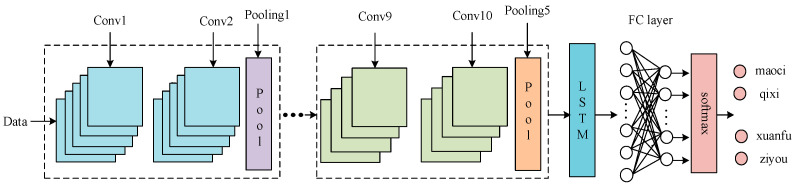
The general framework of convolutional neural network and long short-term memory (CNN-LSTM).

**Figure 4:**
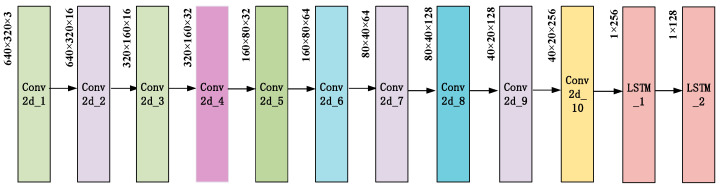
The detailed structure of CNN-LSTM.

When dealing with time-series data, LSTM has unique advantages. However, for a large number of samples, its feature extraction performance is poor, and a lot of calculation time is required to obtain satisfactory results. Based on the above features, this article uses the CNN-LSTM structure. The CNN part uses convolutional layers and pooling for data dimensionality reduction and spatial feature extraction, and the LSTM network further extracts the time features of the data [18,19,20]. As shown in Figure 3 and Figure 4, a CNN module, a LSTM module, and a feature fusion module constitute a CNN-LSTM hybrid module. First, a single-input model is established using four PD time-voltage datasets. In order to reduce the convolution kernel, increase the depth of the network model, and enhance the feature extraction performance, CNN adopts stacked convolution and pooling operations. Then, the eigenvalues are converted into eigenvectors, and the LSTM gate structure is used to effectively extract the time-series features to improve the generalization ability of the model. Finally, feature fusion is performed on the features of the previous layer through the FC connection layer, the data are classified through the softmax layer, and the probability value is outputted to realize the feature recognition of the PD signal.

**Figure 5:**
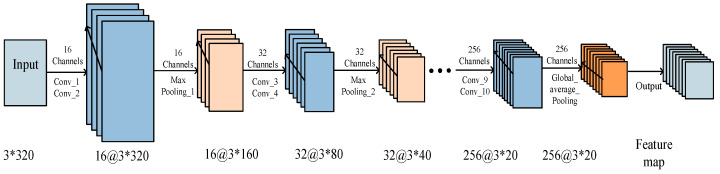
The structure of the CNN in the proposed CNN-LSTM.

As shown in Figure 5, the CNN in the CNN-LSTM hybrid network uses 2D convolution, the shape of the convolution kernel is set to 3 × 3, and the step size is set to 2. The number of single-layer convolution kernels is 16, 32, 64, 128, and 256, and the number of double-layer convolution kernels is the same as the number of convolution kernels in the previous layer, with a total of 10 layers. The pooling layer has five layers, the window size of each layer is 2 × 2, and the step size is 2. The activation function is set to Relu, and the pooling operation of the last layer adopts global average pooling. The number of LSTM layers is set to 2, the number of units is 128 and 64, and the remaining parameters are system default values.

## 4. Experimental Evaluation

### 4.1. Data Acquisition

**Figure 6:**
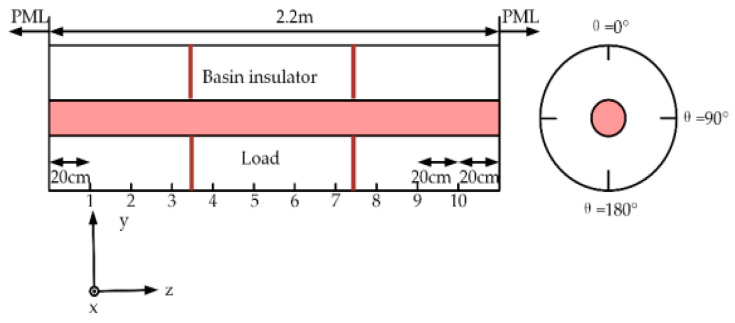
Defect simulation model.

In order to obtain as many fault samples as possible, this paper uses the simulation software XFDTD to simulate the typical defect signals of GIS partial discharge. The four types of fault signals are: metal tip defect (type 0), insulator air gap defect (type 1), floating electrode defect (type 2), free metal particle defect (type 3). The defect simulation model is shown in Figure 6.

**Figure 7:**
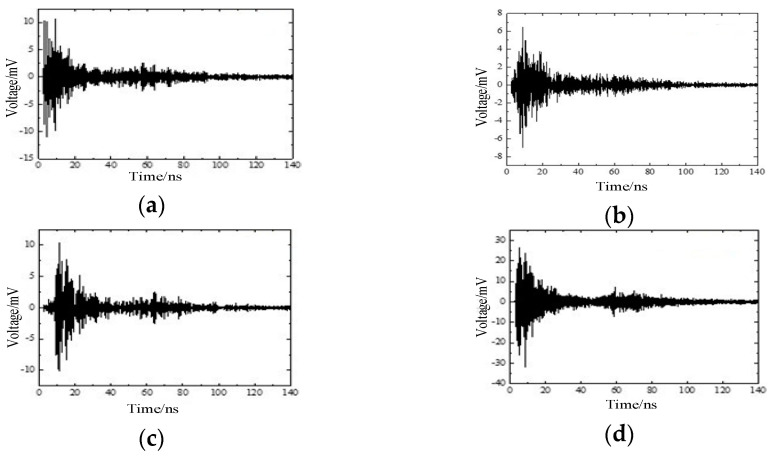
Simulation waveforms under four typical defects: (**a**) metal tip, (**b**) air gap in insulator, (**c**) floating electrode, and (**d**) free metal particle defects.

The simulation adopts the electromagnetic simulation software XFDTD. Import the simulation model established in Creo into XFDTD, as shown in Figure 2. The current pulse signals of four different partial discharge defects are used as the excitation source and imported into the simulation model, and the voltage probes of the 50 Ω load at different positions are set to obtain the required voltage signal. Four types of waveforms are obtained through simulation, namely, (a) metal tip, (b) air gap in insulator, (c) floating electrode, and (d) free metal particle defects, as shown in Figure 7 [21,22]. At the same time, it can be found that the voltage signals corresponding to different partial discharge defects are different, and the difference can be used to realize the identification of different partial discharge defect types.

### 4.2. Training Process

The configuration of the PD pattern recognition device is as follows: Intel i5 processor (2.5 GHz); memory: 16GB; system: win10, running environment: tensorflow = = 2.0.0, keras = = 2.3.0, and python = = 3.7; and recognition object with four typical PD defects of GIS.

From the above simulation, 62 metal tip defect images, 63 air gap defects in the insulator, 64 floating electrode defects, and 63 free metal particles were obtained. A total of 252 images were used as datasets. Among them, 80% of the data samples are used for training, and 20% are used for testing, that is, 201 samples are trained, and 51 samples are verified. The model compilation process uses the cross-entropy loss function, the learning rate is set to 0.0001, the number of epochs is 65, and the batch_size is 32.

**Figure 8:**
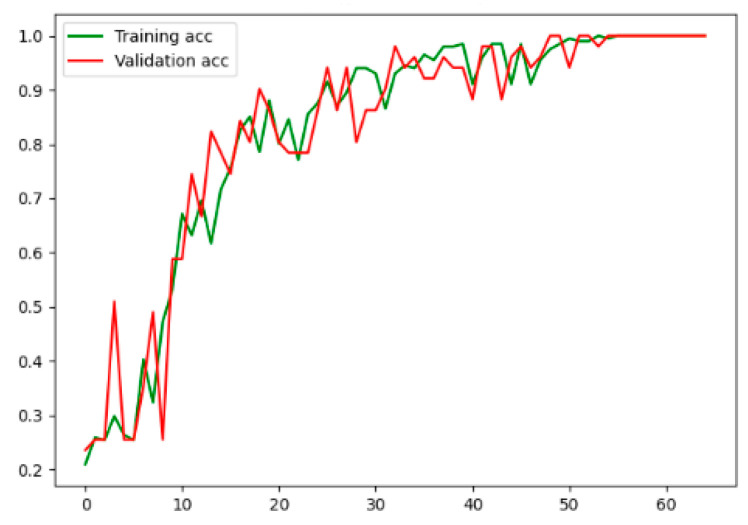
The training accuracy and validation accuracy curves of CNN-LSTM.

The training accuracy and validation accuracy curves during the model training are shown in the figure. It can be seen, in Figure 8, that as the number of training steps increases, the training accuracy gradually increases and then stabilizes, while the validation accuracy generally increases but decreases at some points. The final training accuracy and verification accuracy curves tend to be flat. The model converges to training. Therefore, it is concluded that the CNN-LSTM model performs well in GIS PD pattern recognition.

### 4.3. Results and Analysis

The processed four kinds of PD data are inputted into the CNN-LSTM network for training and recognition, and the overall prediction accuracy is taken as the evaluation parameter of the GIS PD pattern recognition ability.

First introduce the concepts related to TP, TN, FP, and FN. Generally speaking, TP and TN are in the right situation, TP is a positive type, and TN is a negative type. It is inferred that FP divides the wrong into right, while FN divides the right into wrong.

The precision is the proportion of correct predictions that are positive to all predictions that are positive, as shown in Formula (6).

(6) Precision=TP/(TP+FP).

The recall rate is the proportion of the correct prediction to be positive to all the actual positives, as shown in Formula (7).

(7) Recall=TP/(TP+FN).

The F1-score is the arithmetic mean divided by the geometric mean, and the larger the better. Putting Precision and Recall into Formula (8) will find that when the value of F1 is small, true positive is relatively increased, and false is relatively decreased, that is, both precision and recall are relatively increased, that is, F1 weights both precision and recall.

(8) F1=2TP/(2TP+FP+FN).

The precision, the recall rate, and the F1-score of the test set are shown in Table 1. In the first column of Table 1, 0, 1, 2, and 3 represent the type of PD, which represent the metal tip, air gap in insulator, floating electrode, and free metal particle defects, respectively. It can be seen, in the table, that the proposed CNN-LSTM model has the highest recognition rate for type 1, type 2, and type 3, with a recognition rate of 100%, and the lowest recognition rate for type 0, with a recognition rate of 91.6%.

In order to verify that the model is applicable to the division of various data sets, we conducted five times of ten-fold cross-validation. In the process of cross-validation, the data set is divided into ten parts, and nine parts are used in turn as training data and one part is used as the test data for testing. Through each test, the corresponding correctness rate is obtained. Five such tests were carried out, and models 1 to 5 were obtained. Finally, as shown in Table 2, it can be seen that Model_5 has the highest prediction accuracy. Therefore, we choose Model 5 as the best model.

**Figure 9:**
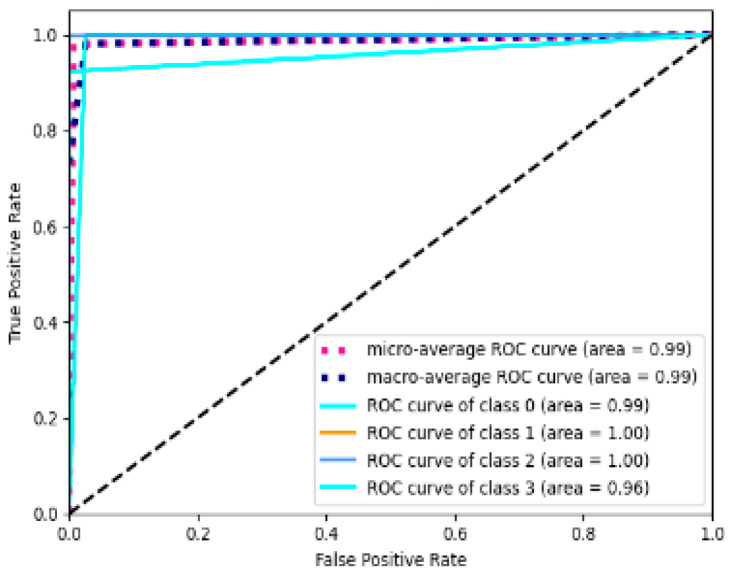
The ROC curve of CNN-LSTM.

The receiver operating characteristic (ROC) curve can provide a view of the overall performance of the classifier. To verify the effect of the proposed model, the ROC curve is used to evaluate the model. The specific results are shown in Figure 9. It can be seen, in Figure 8, that the CNN-LSTM model proposed in this article has no false positives and no missing conditions. The four types of ROC curves are all pushed to the upper left corner, and the area under the curve (AUC) is close to 1. The closer the area is to 1, the better the classification effect. This proves that the CNN-LSTM model proposed in this paper has a good classification performance. As shown in the table below, we performed five times of ten-fold cross-validation to divide the data set into ten parts, and take turns using nine parts as training data and one part as the test data for testing. Through each test, the corresponding correct rate is obtained, and ten such tests were carried out. Finally, the average of the correct rates of these ten results is used as an estimate of the accuracy of the algorithm.

**Figure 10:**
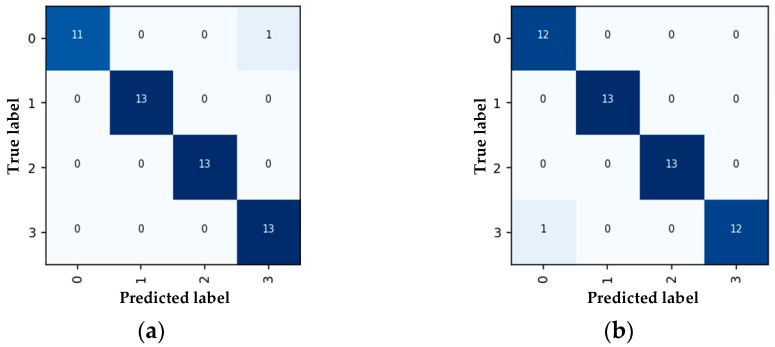
Confusion matrices of CNN-LSTM: (**a**) first training, (**b**) second training.

To visually display the classification effects of the model, a confusion matrix is used to visualize the classification results of the model. The proposed CNN-LSTM model is trained many times, and a confusion matrix appears at the end of each training. Among all the results, Figure 10a,b appears most frequently. Each column of the confusion matrix represents the predicted value, and each row represents the true category. In the confusion matrix (a), there are 12 prediction samples whose true category is type 0, one of which is incorrectly predicted as type 3, and all the remaining samples are predicted correctly. In the confusion matrix (b), there are 13 prediction samples whose true category is type 3, one of which is incorrectly predicted as type 0, and all the remaining samples are predicted correctly. It can be seen that the proposed CNN-LSTM model easily confuses type 0 (metal tip defects) and type 3 (free metal particles defects). However, the model’s recognition accuracy rate for type 0 and type 3 of PD defects still reached 91.6%. Therefore, the four types of PD signals are effectively separated and concentrated in a specific area. The model effectively learns features and realizes data classification.

### 4.4. Comparison of Different Methods

To verify the recognition accuracy of the model, a variety of traditional classification methods were selected for the GIS PD pattern recognition of four types of defects (metal tip, air gap in insulator, floating electrode, and free metal particle defects). When using BPNN, Resnet18, and CNN algorithms, the input data set method is the same, and they are all four types of partial discharge images. The training set accounts for 80% of the total samples, the test set accounts for 20%. The input of LSTM is the coordinate value of time and voltage obtained by XFDTD simulation, that is, the time-voltage coordinate value in the above image is automatically extracted through the simulation software, and then input into the LSTM as text for classification.

**Figure 11:**
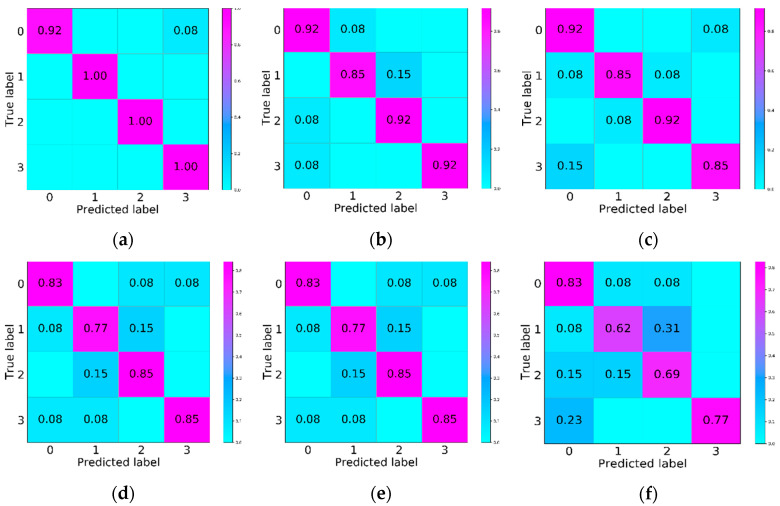
Confusion matrices of different methods: (**a**) CNN-LSTM, (**b**) support vector machines (SVM), (**c**) Resnet18, (**d**) back propagation neural networks (BPNN), (**e**) CNN, and (**f**) LSTM.

The pattern recognition results are shown in Table 2. It can be seen, in Table 2, that the overall recognition rate of the CNN-LSTM model is 97.9%, which is significantly higher than the 92.3% of SVM, 88.3% of Resnet18, 82.4% of BPNN, 82.4% of CNN, and 72.3% of LSTM. It can be seen from the recognition rate of the four types of typical defects that the first type of defect (insulator air gap) has the lowest recognition rate. Small gaps in the insulator or gaps in the layered area between the insulating material and the metal insert will accumulate electric fields, resulting in PD instability [23,24]. The CNN-LSTM proposed in this article can identify 91.6% of type 0 and 100% of type 1, type 2, and type 3 PD defects. Therefore, in general, CNN-LSTM is the best pattern recognition method among the above models. Figure 11 shows the confusion matrices of six typical classification methods. In general, the classification results for type 1 are the most frequently distributed in other regions, and the effect is the most unsatisfactory, which is consistent with the analysis results shown in Table 3.

## 5. Conclusions

In this paper, a hybrid network model based on CNN-LSTM is constructed, which uses time domain and frequency domain features to realize end-to-end GIS PD fault diagnosis. The proposed method is divided into three principal steps. First, the local spatial feature information of PD signals is extracted through the CNN convolutional layer and the pooling layer, and the sequence features of the data are preserved. Then, the LSTM hybrid network is constructed to extract the identification features of all PD signals containing timing features. Finally, the softmax layer is applied for classification. This algorithm combines the advantages of CNN, which is good at mining and extracting spatial features, and LSTM, which is good at mining the time-series feature information of PD maps.

The effectiveness of the algorithm is verified, and the results show that the CNN-LSTM proposed in the article has the highest recognition rate (100%) for type 1, type 2, and type 3 defects, and the lowest recognition rate (91.6%) for type 0 defects, with an average recognition ability of 97.9%. Thus, the algorithm can effectively realize the pattern recognition of PD defects. In addition, compared with other traditional algorithms, the overall recognition rate of the CNN-LSTM model proposed in this article is higher than that of the traditional algorithms listed. Not only can the model effectively extract and use the spatio-temporal features of the input, but it also uses the LSTM structure to enhance the generalization ability of the model. This provides a new framework for GIS PD fault diagnosis. In addition, in the field of unbalanced data distribution in fault diagnosis, we will further investigate the unbalanced distribution of learning data to further improve the performance of the algorithm.

## Figures and Tables

**Table 1:** Training results of CNN-LSTM.

Type	Precision (%)	Recall (%)	F1-Score (%)
0	91.6	100	95.8
1	100	100	100
2	100	100	100
3	100	91.6	95.8

**Table 2:** Result of the models’ 10-fold cross-validation.

Type	Model_1	Model_2	Model_3	Model_4	Model_5
0	91.6	100	91.6	100	100
1	100	92.3	92.3	92.3	100
2	92.3	92.3	100	100	100
3	100	92.3	100	92.3	91.6
Overall	96.0	94.2	96.0	96.2	97.9

**Table 3:** PD recognition results of different methods.

Type	CNN-LSTM	SVM	Resnet18	BPNN	CNN	LSTM
0	91.6	91.6	91.6	83.3	83.3	83.3
1	100	84.6	84.6	76.9	76.9	61.5
2	100	92.3	92.3	84.6	84.6	76.9
3	100	92.3	84.6	84.6	84.6	69.2
Overall	97.9	92.3	88.3	82.4	82.4	72.3

## Data Availability

The data presented in this study are available on request from the corresponding author.
